# The NF-κB p65 and p50 homodimer cooperate with IRF8 to activate iNOS transcription

**DOI:** 10.1186/s12885-015-1808-6

**Published:** 2015-10-23

**Authors:** Priscilla S. Simon, Sarah K. Sharman, Chunwan Lu, Dafeng Yang, Amy V. Paschall, Sidhartha S. Tulachan, Kebin Liu

**Affiliations:** 1 Department of Biochemistry and Molecular Biology, Medical College of Georgia, Georgia Regents University, Augusta, GA 30912 USA; 2 Medicine, Medical College of Georgia, Georgia Regents University, Augusta, GA 30912 USA; 3 Cancer Center, Georgia Regents University, Augusta, GA 30912 USA; 4 Charlie Norwood VA Medical Center, Augusta, GA 30904 USA

**Keywords:** iNOS, NF-κB, IFNγ, IRF8, pSTAT1, Colon cancer

## Abstract

**Background:**

Inducible nitric oxide synthase (iNOS) metabolizes L-arginine to produce nitric oxide (NO) which was originally identified in myeloid cells as a host defense mechanism against pathogens. Recent studies, however, have revealed that iNOS is often induced in tumor cells and myeloid cells in the tumor microenvironment. Compelling experimental data have shown that iNOS promotes tumor development in certain cellular context and suppresses tumor development in other cellular conditions. The molecular mechanisms underlying these contrasting functions of iNOS is unknown. Because iNOS is often induced by inflammatory signals, it is therefore likely that these contrasting functions of iNOS could be controlled by the inflammatory signaling pathways, which remains to be determined.

**Methods:**

iNOS is expressed in colon carcinoma and myeloid cells in the tumor microenvironment. Colon carcinoma and myeloid cell lines were used to elucidate the molecular mechanisms underlying iNOS expression. Chromatin immunoprecipitation and electrophoretic mobility shift assay were used to determine the IFNγ-activated pSTAT1 and NF-κB association with the chromatin DNA of the *nos2* promoter.

**Results:**

We show here that iNOS is dramatically up-regulated in inflammed human colon tissues and in human colon carcinoma as compared to normal colon tissue. iNOS is expressed in either the colon carcinoma cells or immune cells within the tumor microenvironment. On the molecular level, the proinflammatory IFNγ and NF-κB signals induce iNOS expression in human colon cancer cells. We further demonstrate that NF-κB directly binds to the *NOS2* promoter to regulate iNOS expression. Although neither the IFNγ signaling pathway nor the NF-κB signaling pathway alone is sufficient to induce iNOS expression in myeloid cells, IFNγ and NF-κB synergistically induce iNOS expression in myeloid cells. Furthermore, we determine that IFNγ up-regulates IRF8 expression to augment NF-κB induction of iNOS expression. More interestingly, we observed that the p65/p65 and p50/p50 homodimers, not the canonical p65/p50 heterodimer, directly binds to the *nos2* promoter to regulate iNOS expression in myeloid cells.

**Conclusions:**

IFNγ-induced IRF8 acts in concert with NF-κB to regulate iNOS expression in both colon carcinoma and myeloid cells. In myeloid cells, the NF-κB complexes that bind to the *nos2* promoter are p65/p65 and p50/p50 homodimers.

## Background

Nitric Oxide Synthase (NOS) metabolizes L-arginine to form the intermediate OH-L-arginine, which is then oxidized into nitric oxide (NO) and L-citrulline in eukaryotic cells. Inducible NOS (iNOS, also termed NOS2) is a type of NOS which was originally identified in myeloid cells. iNOS is induced in myeloid cells after activation by endotoxins or cytokines to generate NO that acts as a defense effector to suppress invading microorganisms or neoplastic tissue [1, 2]. However, it is now clear that iNOS is also present in numerous types of non-immune cells, including endothelial cells, fibroblasts, vascular smooth muscle cells, cardiac myocytes, and cancerous cells [3, 4].

Consistent with its function as a host defense agent, iNOS can protect certain host tissues from certain infectious diseases. Compelling experimental data indicate that iNOS is inducible in tumor cells and function as a tumor suppressor [3–9]. NO functions as a cytotoxic agent that can suppress cancer development [10–15]. However, overwhelming experimental data from both human cancer patients and mouse tumor models indicate that iNOS can also promote tumor development [16–25]. It is now clear that iNOS induction is a common phenomenon of chronic inflammation, and iNOS-produced NO acts as a key signaling molecule that promotes inflammation-mediated spontaneous colon cancer development [18, 26]. One mechanism underlying iNOS function in tumor promotion might be its function in regulation of the tumor-initiating properties of cancer stem cells [27, 28].

Myeloid cells are often abundantly present in many solid tumors [29], and are another major site of iNOS expression [8, 9, 30]. Like in tumor cells, iNOS also exhibits contrasting functions in myeloid cells in the context of tumor development [18]. It has been shown that macrophages are required for phthisic rejection of intraocular tumors, and *in vitro* and *in vivo* inhibition of iNOS abolished macrophage-mediated killing of tumor cells and rejection of tumors [2, 8]. Furthermore, under hypoxic conditions, the induction of iNOS activity in myeloid cells is associated with a substantial increase in tumor cell toxicity [9]. However, recent studies suggest that iNOS expressed in myeloid cells also plays a key role in myeloid cell-mediated immune suppression and tumor promotion [31–34]. Myeloid cells from colon carcinoma-bearing mice exhibit elevated iNOS and NO, which is associated with increased levels of nitration on STAT1, resulting in suppression of the anti-tumor immune response [30]. Therefore, iNOS expression occurs in both tumor cells and tumor-associated myeloid cells, and can act in concert to promote tumor development.

iNOS expression is induced by various inflammatory stimuli that activate distinct signaling pathways that converge to initiate expression of iNOS [35, 36]. One of the well-known iNOS inducers is NF-κB [37]. However, NF-κB has contrasting functions as well. NF-κB is a well-documented inflammatory factor that promotes inflammation-mediated colon cancer progression [38, 39]. Overwhelming experimental data also demonstrate that NF-κB is an apoptosis promoter and tumor suppressor [40–44]. These contrasting functions of NF-κB are believed to be cellular context-dependent. The molecular mechanisms underlying NF-κB function in iNOS induction in colon cancer and myeloid cells are still not fully understood. IFNγ is a key component of the host cancer immune surveillance system [45]. However, IFNγ is also a two-edged sword and an inflammatory cytokine that regulates iNOS expression. Chronic IFNγ signaling promotes spontaneous colon cancer development through an iNOS-dependent mechanism [26]. The fact that iNOS functions both to promote and suppress tumor development and that iNOS inducers IFNγ and NF-κB also exhibit both tumor promotion and suppression functions raise the possibility that IFNγ and NF-κB-mediated iNOS induction mechanisms dictate iNOS expression level and functionalities. However, the molecular mechanism underlying IFNγ and NF-κB regulation of iNOS expression is still elusive.

We report here that iNOS is expressed in both human colon carcinoma cells and tumor-infiltrating immune cells. We determined that IFNγ and NF-κB synergistically induce iNOS expression in both tumor cells and myeloid cells. Furthermore, IFNγ up-regulates IRF8 expression that is essential for IFNγ and NF-κB induction of iNOS expression. We determined that NF-κB functions through direct binding to the iNOS promoter to activate iNOS transcription. In myeloid cells, the p65/p65 and p50/p50 NF-κB homodimers directly bind to the iNOS promoter, thereby revealing an essential role of the p65/p65 and p50/p50 homodimers in NF-κB induction of iNOS.

## Methods

### Cell lines and human tissue specimens

The human colon carcinoma T84 cell lines and murine J774 myeloid cell lines were obtained from American Type Culture Collection (ATCC) (Manassas, VA). ATCC has characterized these cells by morphology, immunology, DNA fingerprinting, and cytogenetics. The CL-2 cell line was kindly provided by Dr. Keiko Ozato (National Institutes of Health, Bethesda, MD) [46]. De-identified human colon carcinoma specimens were obtained from the Cooperative Human Tissue Network and used with approval by The Georgia Regents University Human Assurance Committee (approval # 730193–1).

### RT-PCR analysis

Total RNA was isolated from cells using Trizol (Invitrogen, San Diego, CA) according to the manufacturer’s instructions, and used for cDNA synthesis using the MMLV reverse transcriptase (Promega, Madison, WI). The cDNA was then used as the template for PCR amplification. RT-PCR was conducted as previously described [47]. The sequences of primers are listed in Table 1.

**Table 1:** Oligo sequences

Oligo Name	Use	Forward	Reverse
hiNOS	RT-PCR	5'- ACATCACCACACCCCCAACC -3'	5'- GAAAGCAGGAAGCCAGCAGAC -3'
hICSBP (IRF8)	RT-PCR	5'-CCAGATTTTGAGGAAGTGACGGAC-3'	5'-TGGGAGAATGCTGAATGGTGC-3'
hβ-actin	RT-PCR	5'- GGAACGGTGAAGGTGACAGCAG -3'	5'- TGTGGACTTGGGAGAGGACTGG -3'
hiNOS-ChIP1	Chromatin immunoprecipitation	5'- CCACAGGTCAAGAATGCCACAC -3'	5'- AATGCCCCCACCCAAGAGCC -3'
hiNOS-ChIP2	Chromatin immunoprecipitation	5'- ACTCCTAATCATCCCTCAAAACCC -3'	5'- CATCTGCCACGAAGAGCAATG -3'
hiNOS-ChIP3	Chromatin immunoprecipitation	5'- GGACTTGGGACCAGAAAGAGGTG -3'	5'- GCCATCCAGAGAGTTGTTTTTGC -3'
hiNOS-ChIP4	Chromatin immunoprecipitation	5'- GGTCTCTTCCTGGTTTGACTGTCC -3'	5'- TTCCAACACCTTCTCTCTGTAGGC -3'
hiNOSNF-κB Probe	EMSA	5'-AAAATTGTGGGAATTTTCTGCCTAC-3'	5'-GTAGGCAGAAAATTCCCACAATTTT-3'
NFB WT Probe	EMSA	5'-CGGGAATTCCC-3'	5'-GGGAATTCCCG-3'
miNOS	RT-PCR	5'-CCAGAGGACCCAGAGACAAGC-3'	5'-GGCAGCACATCAAAGCGGC-3'
mβ-actin	RT-PCR	5'-CTGGCACCACACCTTCTACAATG-3'	5'-GGGTCATCTTTTCACGGTTGG-3'
miNOSChIP1	Chromatin immunoprecipitation	5'-ATGGTGTCTTCTGCCTCGCAAG-3'	5'-CCCCAGGATTCCACTGTTGAAC-3'
miNOSChIP2	Chromatin immunoprecipitation	5'-AAAGGAGAAACAGCCACCAAGC-3'	5'-AGCACCCACAACCCAAAGAAC-3'
miNOSChIP3	Chromatin immunoprecipitation	5'-TCCATCCCCTGAGCAATGTG-3'	5'-CCCCCCAAACCCAATACTTG-3'
miNOSChIP4	Chromatin immunoprecipitation	5'-CACAGCCCATCCACTATTCTGC-3'	5'-CCAGGACACATTCATCAGGAGG-3'
miNOSChIP5	Chromatin immunoprecipitation	5'-ACTCAGGGTAGGGTCCAGTTCATC-3'	5'-TATGTGGCTTCTCCTTGGCGAG-3'
miNOSNF-κB Probe	EMSA	5'-GCTAGGGGGATTTTCCCTCTCTC-3'	5'-GAGAGAGGGAAAATCCCCCTAGC-3'

### Immunoprecipitation and Western blotting analysis

Western blotting analysis was performed as previously described [48]. The blot was probed with antibodies specific for iNOS (BD Biosciences), STAT1 (BD Biosciences), pSTAT1 (BD Biosciences), and β-actin (Sigma-Aldrich). Immunoprecipitation was done with anti-p65 and anti-p50 (Santa Cruz Biotech), as previously described [49, 50]. The immunoprecipitated proteins were analyzed by Western blot analysis with anti-p65 (Santa Cruz Biotech).

### Cell treatment

Cells were treated with IFNγ (100 IU/ml, PeproTech), TNFα (100 IU/ml, R & D System), and Lipopolysaccharide (LPS, 1 μg/ml, Sigma-Aldrich) as indicated overnight. Jak-STAT inhibitor Ruxolitinib (250 nM, LC Laboratories) was added to the cell culture 30 min before addition of IFNγ, TNFα, or LPS.

### Immunohistochemistry

Immunohistochemical staining was performed at the Georgia Pathology Service. iNOS-specific antibody was obtained from Santa Cruz Biotech.

### Chromatin immunoprecipitation (ChIP) assay

ChIP assays were carried out using anti-p65 and anti-p50 antibodies (Santa Cruz Biotech) and protein A-agarose beads (Millipore) as previously described [48]. The human and mouse iNOS promoter DNA was detected by PCR using gene-specific primers (Table 1).

### Protein-DNA interaction assay

DNA-protein interaction was determined by electrophoresis mobility shift assay (EMSA) as previously described [51]. Nuclear extracts were prepared as previously described [52]. The probe sequences are listed in Table 1.

### Gene silencing

Tumor cells were transiently transfected with scramble and IRF8-specific siRNAs (Santa Cruz Biotech), respectively and analyzed for IRF8 and iNOS expression by RT-PCR.

### Gene overexpression

Cells were electroporated with pcDNA 3.1 (vector control) or pcDNA.IκBa-AA (kindly provided by Dr. Michael Karin, University of California, San Diego). The cells were then cultured overnight and treated with IFNγ and LPS for another 18 h.

## Results

### iNOS expression profiles in human colon tissues

**Fig. 1:**
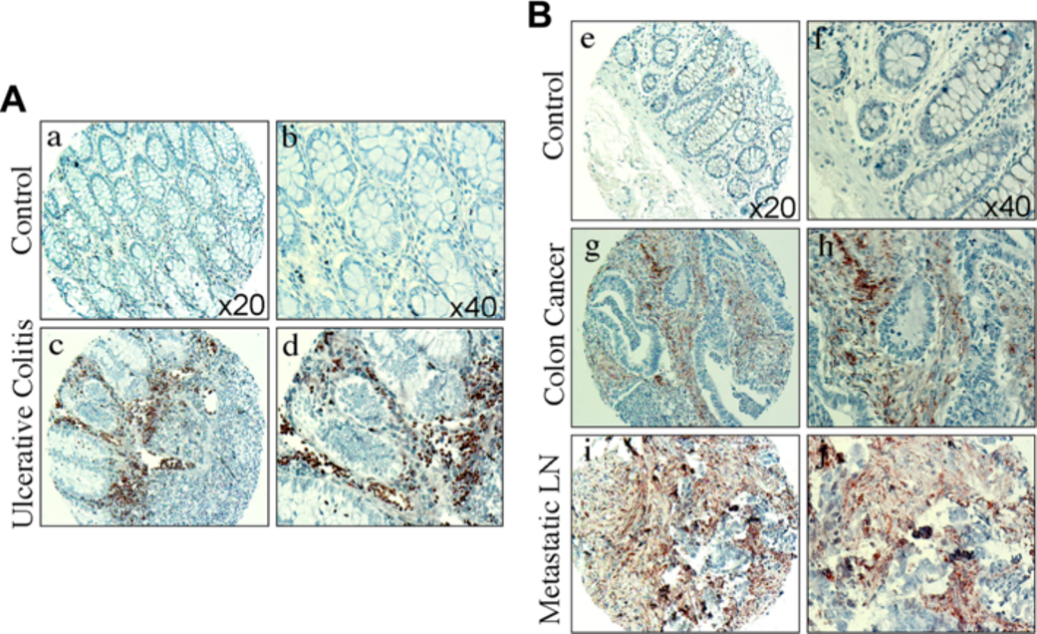
iNOS protein profiles in human colon tissues. **a** Normal colon tissues (*a* & *b*) and colon tissues from human ulcerative colitis patients (*c* & *d*) were stained with iNOS-specific antibody. Shown are images of representative results. Upregulation of iNOS expression was seen in inflammatory cells of ulcerative colitis (*c* & *d*). **b** Normal colon tissues (*e* & *f*), primary colon carcinoma tissues (*g* & *h*) and Metastatic Lymph Node (LN) (*i* & *j*) were stained with iNOS-specific antibody. Shown are images of representative results. Normal colon tissues exhibit no detectable iNOS (*e* & *f*). Upregulation of iNOS expression was seen in inflammatory cells of the primary tumor tissues (*g* & *h*) and Metastatic LN (*i* & *j*)

We made use of a human colorectal cancer tissue microarray (Cooperative Human Tissue Network) and stained for iNOS protein levels. Because colonic inflammation is a key cause of colon cancer, we focused our analysis on colon tissues from human ulcerative colitis patients and colon cancer patients. The normal human colon tissues exhibit no detectable iNOS protein level. In contrast, iNOS protein level is high in colon tissues from ulcerative colitis patients (Fig. 1a). Human primary colon carcinoma tissues also exhibit high level of iNOS, but most of the iNOS-positive cells are non-tumor cells in the tumor microenvironment (Fig. 1b). However, the lymph node metastatic colon carcinoma cells exhibit high iNOS expression level (Fig. 1b). These observations indicate that iNOS is up-regulated in inflammatory colon epithelial cells, colon carcinoma cells, and tumor-infiltrating immune cells.

### iNOS expression patterns in the human tumor microenvironment.

**Fig. 2:**
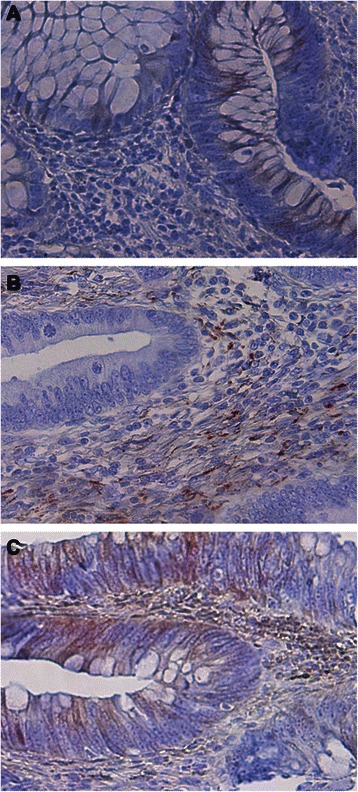
iNOS protein level in human colon carcinoma tissues. Human colon carcinoma specimens were stained with iNOS-specific antibody. Shown are images of iNOS protein in tumor cells only (**a**), in tumor-infiltrating immune cells only (**b**), and in both tumor cells and tumor-infiltrating immune cells (**c**)

Further analysis of the primary human colon carcinoma tissues revealed three types of iNOS expression patterns: 1) only tumor cells are iNOS-positive and no adjacent immune cells contain iNOS (Fig. 2a); 2) tumor cells exhibit undetectable iNOS, but adjacent immune cells express iNOS (Fig. 2b); and 3) both tumor cells and adjacent immune cells have detectable iNOS protein (Fig. 2c). These observations indicate that iNOS is expressed in both colon cancer cells and tumor-infiltrating immune cells under pathological conditions.

### IFNγ and TNFα synergistically induce iNOS expression in human colon carcinoma cells.

**Fig. 3:**
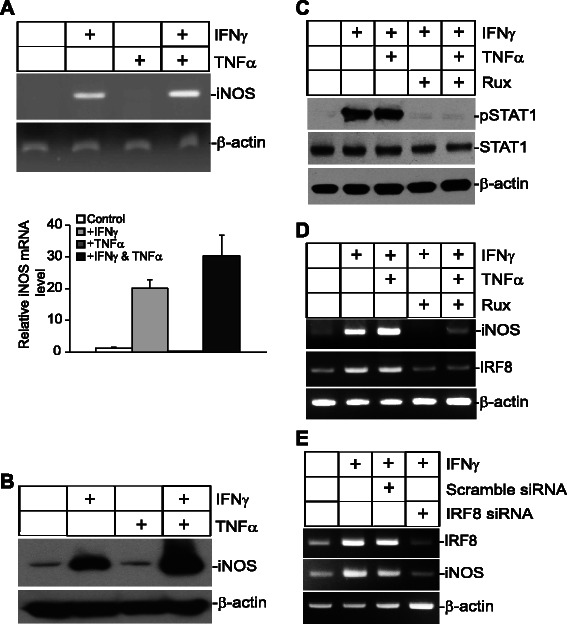
IFNγ and TNFα cooperatively induce iNOS expression in human colon carcinoma cells. **a** Tumor cells were treated with IFNγ, TNFα, or both IFNγ and TNFα for approximately 18 h, and analyzed for iNOS expression by RT-PCR. β-actin was used as a normalization control. **b** Cells were treated as in A and then analyzed by Western blotting analysis of iNOS expression with β-action as an internal control. **c** Tumor cells were cultured in the presence of Ruxolitinib for 30 min and then treated with IFNγ and TNFα as indicated for 18 h. Total lysates were then prepared and analyzed for STAT1 and pSTAT1 levels by Western blotting analysis. **d** The cells were treated as in C and then analyzed by RT-PCR for iNOS expression. **e** The cells were transfected with either scramble siRNA or human IRF8-specific siRNA for 6 h and the cells were treated with IFNγ for 18 h. The cells were analyzed for IRF8 and iNOS expression by RT-PCR with β-actin as a normalization control

The IFNγ and NF-κB signaling pathways have been shown to regulate iNOS expression in various types of cells [35, 36, 53–57]. To elucidate the molecular mechanisms underlying IFNγ- and NF-κB-mediated iNOS expression in human colon carcinoma cells, human colon carcinoma T84 cells were treated with IFNγ, TNFα, or both IFNγ and TNFα, and then analyzed for iNOS expression. RT-PCR analysis revealed that, as expected, IFNγ induced iNOS expression in T84 cells. TNFα alone did not induce iNOS expression (Fig. 3a), but TNFα dramatically increased IFNγ-induced iNOS expression (Fig. 3a). Consistent with iNOS mRNA expression patterns, iNOS protein levels were dramatically increased by IFNγ but not by TNFα treatment (Fig. 3b). However, TNFα dramatically increased IFNγ-induced iNOS expression (Fig. 3b).

To determine that IFNγ specifically induces iNOS expression, tumor cells were cultured in the presence of Ruxolitinib, a specific Jak/STAT inhibitor. As expected, Ruxolitinib blocked IFNγ mediated STAT1 activation in human colon carcinoma cells (Fig. 3c). Consistent with inhibition of STAT1 activation, Ruxolitinib inhibited IFNγ induction of iNOS expression in the tumor cells (Fig. 3d). Interestingly, Ruxolitinib also diminished TNFα function in enhancing IFNγ induction of iNOS expression (Fig. 3d), suggesting that both the IFNγ signaling pathway and NF-κB are essential for iNOS induction. It has been previously reported that iNOS is regulated by IRF8 [58]. It is also known that IRF8 is regulated by IFNγ-activated pSTAT1 [59]. Therefore, we reason that IFNγ activates pSTAT1 to activate IRF8 to upregulate iNOS. To test this hypothesis, we treated T84 cells with IFNγ and TNFα in the presence of Ruxolitinib and analyzed IRF8 expression. Indeed, Ruxolitinib inhibited IRF8 expression. In order to validate the above observation that IFNγ regulates iNOS expression through IRF8, a complimentary approach was used to determine the relationship between IRF8 and iNOS. IRF8 was silenced in T84 cells using an IRF8-specific siRNA. RT-PCR analysis indicate that silencing IRF8 expression diminished IFNγ-induced iNOS expression (Fig. 3e).

### NF-κB binds to the *NOS2* promoter to regulate iNOS expression

**Fig. 4:**
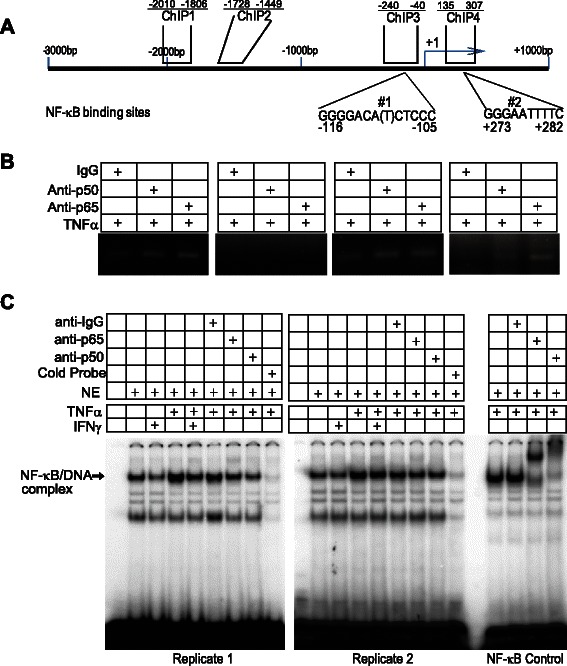
NF-κB binds to NOS2 promoter to activate iNOS expression in human colon carcinoma cells. **a** The *NOS2* gene promoter structure. The NF-κB consensus sequences are indicated. The locations of ChIP PCR primers are also indicated. **b** ChIP analysis of NF-κB association with the *NOS2* gene promoter. **c** EMSA of NF-κB binding to DNA. Human colon carcinoma cells were treated as indicated and used for nuclear extract preparation. The DNA probes containing the NF-κB consensus sequence (#2 as shown in A) were incubated with the nuclear extracts and analyzed for NF-κB-DNA association. Shown are duplicated results (Replicate 1 and Replicate 2). A NF-κB control probe (Santa Cruz Biotech) was used as a positive control probe. The probe sequences are presented in Table 1

IFNγ activates gene expression through Jak-dependent activation of STAT1 that binds to the target gene promoters to activate transcription [53], whereas TNFα activates NF-κB to directly activate gene transcription [35, 60]. The above results indicate that pSTAT1 indirectly regulates iNOS through IRF8 (Fig. 3e). Analysis of the human *NOS2* gene promoter identified two putative NF-κB-binding consensus sequences (Fig. 4a). To determine whether TNFα-activated NF-κB directly bind to the *NOS2* promoter to activate iNOS transcription, NF-κB-specific antibodies were used to determine the interactions of NF-κB with the *NOS2* promoter chromatin. NF-κB association with the NOS2 promoter chromatin was detected in three regions of the *NOS2* promoter region in TNFα-treated tumor cells (Fig. 4b). To validate these findings, oligonucleotides containing the NF-κB-binding consensus sequences of the *NOS2* promoter (Fig. 4a) were synthesized. Oligonucleotides were annealed to generate double-stranded DNA probe (Table 1). The probes were labeled with ^32^P and incubated with nuclear extracts prepared from untreated and treated cells. These DNA-protein interactions were analyzed by EMSA. Specific NF-κB/DNA interactions were detected, indicated that NF-κB directly regulates iNOS expression (Fig. 4c).

### pSTAT1 and NF-κB synergistically regulate iNOS expression in myeloid cells

**Fig. 5:**
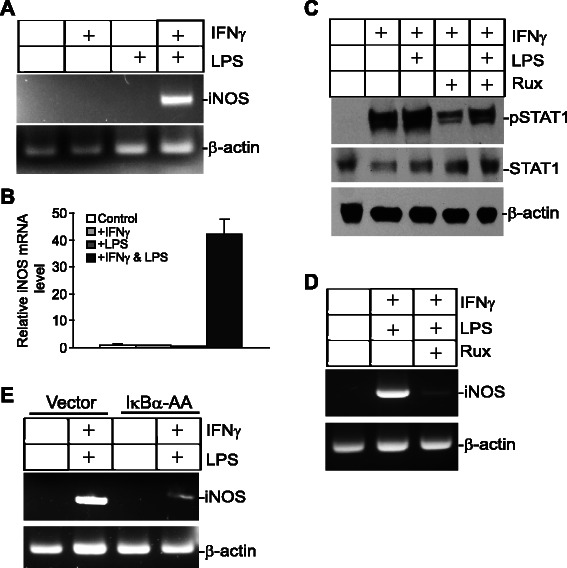
IFNγ and NF-κB induce iNOS expression in myeloid cells. **a** J774 cells were treated with IFNγ, LPS, or both IFNγ and LPS for approximately 18 h, and analyzed for iNOS expression by RT-PCR. β-actin was used as a normalization control. **b** Cells were treated as in A and then analyzed by real time RT-PCR analysis of iNOS expression with β-action as an internal control. **c** J774 cells were cultured in the presence of Ruxolitinib for 30 min and then treated with IFNγ and LPS as indicated for 18 h. Total lysates were then prepared and analyzed for STAT1 and pSTAT1 levels by Western blotting analysis. **d** J774 cells were cultured in the presence of Ruxolitinib for 30 min and then treated with IFNγ and LPS for 18 h. iNOS expression was then analyzed by RT-PCR. **e** J774 cells were transiently transfected with a control vector or a vector containing the dominant negative IκBα-AA mutant, respectively. Cells were treated with IFNγ and LPS for approximately 18 h, and then analyzed for iNOS expression

To determine whether pSTAT1 and NF-κB also cooperate to up-regulate iNOS expression in myeloid cells, myeloid J774 cells were treated with IFNγ, LPS, or both IFNγ and LPS. RT-PCR analysis revealed that neither IFNγ nor LPS alone is sufficient to induce iNOS expression. However, combined IFNγ and LPS dramatically induced iNOS expression in J774 cells (Fig. 5a & b). To determine whether IFNγ increases iNOS expression specifically through the Jak-STAT signaling pathway, J774 cells were cultured in the presence of Ruxolitinib prior to IFNγ and LPS treatment. As expected, Ruxolitinib inhibited IFNγ-induced STAT1 activation (Fig. 5c). It is also clear that Ruxolitinib specifically inhibits NF-κB and IFNγ-mediated iNOS expression induction (Fig. 5d). Next, we transiently transfected J774 cells with a IκBα-AA plasmid, a dominant-negative mutant of IκBα that blocks activation of the canonical NF-κB. RT-PCR analysis revealed that blocking NF-κB activation inhibited IFNγ and LPS-induced iNOS expression in J774 cells (Fig. 5e). These observations indicate that the IFNγ-activated Jak-STAT signaling pathway acts in concert with NF-κB to regulate iNOS expression in myeloid cells.

### The NF-κB homodimers bind to the *nos2* promoter to regulate iNOS expression

**Fig. 6:**
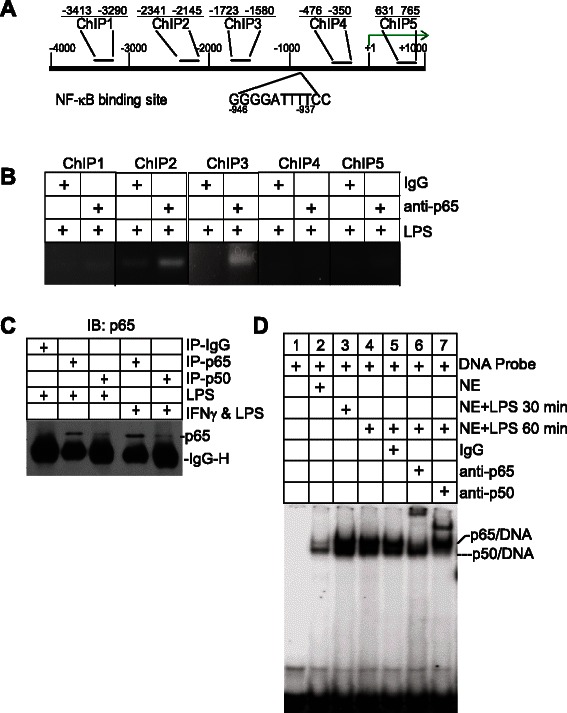
NF-κB binds to *nos2* promoter to activate iNOS expression in myeloid cells. **a** The *nos2* gene promoter structure. The NF-κB consensus sequence is indicated. The locations of ChIP PCR primers are also indicated. **b** ChIP analysis of NF-κB association with the *nos2* gene promoter. **c** J774 cells were treated with LPS for approximately 1 h. Nuclear extracts were prepared from the cells and used for immunoprecipitation (IP) with anti-p65 and anti-p50 antibodies, respectively. The IP was then analyzed by Western blotting analysis using p65-specific antibody. **d** EMSA of NF-κB binding to *nos2* promoter DNA. J774 cells were treated as indicated and used for nuclear extract preparation. The DNA probe containing the NF-κB consensus sequence as shown in A was incubated with the nuclear extracts and analyzed for NF-κB-DNA association using p65- and p50-specific antibodies

Analysis of the mouse *nos2* gene promoter region identified a putative NF-κB-binding consensus sequence (Fig. 6a). ChIP was then used to determine whether NF-κB directly binds to the *nos2* promoter chromatin. NF-κB directly binds to multiple sites on the nos2 promoter region in LPS-treated J774 cells (Fig. 6b).

NF-κB contains 5 Rel subunits, and the most common NF-κB dimer is the p65/p50 heterodimer. IP-Western blotting analysis revealed that indeed the p65/p50 NF-κB heterodimer exists in LPS-treated and both LPS and IFNγ-treated J774 cells (Fig. 6c). To validate that NF-κB exists as the p65/p50 heterodimer at the *nos2* promoter region, we performed EMSA assays using p65- and p50-specific antibodies. The rationale is that if NF-κB binds to the NF-κB consensus sequence DNA of the *nos2* promoter, then p65/p50-DNA complexes should be detected. Analysis of protein-DNA interactions with nuclear extracts from LPS-treated J774 cells and the *nos2* promoter NF-κB consensus sequence-containing DNA probe identified two protein-DNA complexes (Fig. 6d). Surprisingly, p65- and p50-specific antibody supershifts revealed that one of the DNA-protein complexes is the p65/p65-DNA complex and another is the p50/p50-DNA complex. No p65/p50-DNA complex was detected. Therefore, the p65/p65 and p50/p50 homodimers, not the p65/p50 heterodimer, bind to the *nos2* promoter region directly in myeloid cells.

### IFNγ up-regulates IRF8 to enhance NF-κB-induced iNOS expression.

**Fig. 7:**
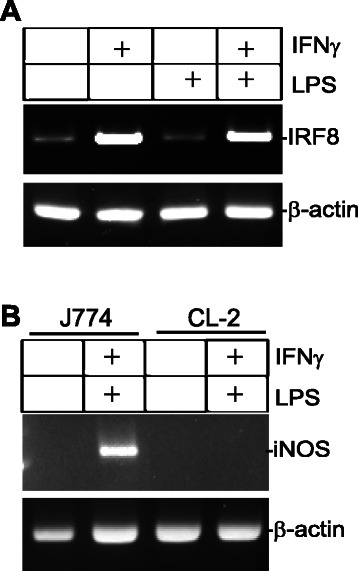
IFNγ up-regulates IRF8 expression to enhance NF-κB-activated iNOS expression. **a** J774 cells were treated with IFNγ and LPS as indicated for approximately 18 h and analyzed for IRF8 expression by RT-PCR. β-actin was used as a normalization control. **b** J774 and CL-2 cells were treated with IFNγ and LPS for 18 h and analyzed for iNOS expression by RT-PCR. β-actin was used as a normalization control

Because pSTAT1 enhances iNOS expression through the intermediate factor IRF8 in human colon carcinoma cells (Fig. 3d & e), we hypothesized that IFNγ activates IRF8 to regulate iNOS expression in myeloid cells as well. To test this hypothesis, we first analyzed IRF8 expression. As expected, IFNγ treatment dramatically increased IRF8 expression [59] (Fig. 7a), and LPS alone did not induce IRF8 expression (Fig. 7a). We then analyzed iNOS induction in IRF8-deficient cells. The rationale is that if IRF8 is essential for IFNγ and NF-κB-mediated iNOS expression, then IRF8 deficiency should cause the loss of iNOS induction by IFNγ and LPS. The IRF8 wild type J774 cells and the IRF8-deficient CL-2 cells [46] were treated with IFNγ and LPS. RT-PCR analysis revealed that iNOS is induced in J774 but not in the IRF8-deficient CL-2 cells (Fig. 7b). Therefore, we, conclude that NF-κB enhances the IFNγ-IRF8 axis-mediated induction of iNOS expression in myeloid cells.

## Discussion

The expression of iNOS is induced by extracellular stimuli that activate distinct signaling pathways that converge to regulate iNOS transcription. Among the various extracellular stimuli, LPS, TNFα, and IFNγ are the three most extensively studied iNOS inducers [1, 35, 36, 53, 54, 56, 57, 61, 62]. IFNγ functions through activating the Jak-STAT signaling pathway, whereas LPS and TNFα induce NF-κB activation to activate iNOS transcription. We show here that iNOS is expressed in both colon carcinoma cells and the tumor-infiltrating immune cells in the tumor microenvironment of human colon carcinoma tissues *in vivo*. Using the human colon carcinoma T84 cell line and the murine myeloid J774 cell line as *in vitro* model systems, we observed that both IFNγ-activated Jak-STAT and LPS/TNFα-activated NF-κB are essential for iNOS induction in both the tumor cells and the myeloid cells. Furthermore, the Jak-STAT signaling pathway and NF-κB synergistically activate iNOS transcription. Strikingly, although LPS induces the canonical p65/p50 heterodimer activation and nuclear translocation (Fig. 6c), no p65/p50-iNOS promoter DNA interactions are detected in myeloid cells. Instead, we observed that the p65/p65 and p50/p50 homodimers bind to the iNOS promoter (Fig. 6d). Our data thus reveal a novel finding that the NF-κB p65/p65 and p50/p50 homodimers, not the canonical p65/p50 heterodimers, directly bind to the NF-κB consensus sequence element at the *nos2* promoter in myeloid cells.

The transcriptional regulation of iNOS has been the subject of extensive studies due to its diverse mechanisms of regulation. IFNγ is a potent inducer of iNOS in various types of cells. Although IFNγ can activate STAT1 that directly binds to gene promoter DNA to regulate IFNγ target gene transcription, pSTAT1 often regulates IFNγ target gene expression through activating transcription of IFN regulatory factors, including IRF8. IRF8 is a transcription factor that has been shown to regulate iNOS expression [57]. Indeed, IRF8 is dramatically up-regulated by IFNγ in myeloid J774 cells, and loss of IRF8 expression abolished IFNγ function in iNOS induction. Therefore, we conclude that IFNγ induces IRF8 expression to regulate iNOS expression.

For NF-κB-mediated iNOS transcription activation, previous studies have identified several NF-κB-binding consensus sequence elements in both the human and mouse iNOS gene promoter regions [63]. We identified three NF-κB-binding sites in the iNOS promoter region in human colon carcinoma cells and one in mouse myeloid cells. We further demonstrated that NF-κB directly binds to the NF-κB-binding consensus sequences in the iNOS gene promoter regions. Therefore, unlike IFNγ-activated pSTAT1, NF-κB directly binds to the iNOS promoter region to activate iNOS gene transcription in both human colon carcinoma and murine myeloid cells.

## Conclusions

**Fig. 8:**
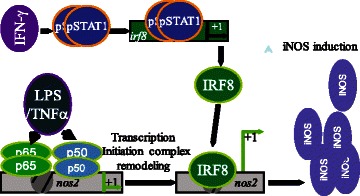
Model of IFNγ and NF-κB action in induction of iNOS expression in myeloid cells. IFNγ and NF-κB induces iNOS expression in tumor cells and myeloid cells. Both IFNγ-activated pSTAT1 and NF-κB are essential for the induction of iNOS. However, IFNγ-activated pSTAT1 does not directly bind to the *nos2* gene promoter. Instead, it activates IRF8 to regulate iNOS transcription. On the other hand, NF-κB directly binds to the *nos2* gene promoter to activate iNOS transcription and it is the p65/p65 and p50/p50 NF-κB homodimers, not the canonical p65/p50 heterodimer, that bind to the *nos2* promoter region to activate iNOS transcription in myeloid cells

Our results provide a novel insight into the molecular mechanisms underlying transcription activation of iNOS gene by IFNγ and NF-κB. IFNγ and NF-κB induces iNOS expression in tumor cells and myeloid cells. Both IFNγ-activated pSTAT1 and NF-κB are essential for the synergistic induction of iNOS. However, IFNγ-activated pSTAT1 does not directly bind to the iNOS gene promoter. Instead, it activates IRF8 to regulate iNOS transcription. On the other hand, NF-κB directly binds to the iNOS gene promoter to activate iNOS transcription. It is the p65/p65 and p50/p50 NF-κB homodimers, not the canonical p65/p50 heterodimer, that bind to the iNOS promoter region to activate iNOS gene transcription in myeloid cells (Fig. 8).
